# Historical Indigenous Food Preparation Using Produce of the Three Sisters Intercropping System

**DOI:** 10.3390/foods10030524

**Published:** 2021-03-03

**Authors:** Tania M. Ngapo, Pauline Bilodeau, Yves Arcand, Marie Thérèse Charles, Axel Diederichsen, Isabelle Germain, Qiang Liu, Shawna MacKinnon, Aimé J. Messiga, Martin Mondor, Sébastien Villeneuve, Noura Ziadi, Stéphane Gariépy

**Affiliations:** 1 Saint-Hyacinthe Research and Development Centre, Agriculture and Agri-Food Canada, 3600 Boulevard Casavant Ouest, Saint Hyacinthe, QC J2S 8E3, Canada; yves.arcand@canada.ca (Y.A.); isabelle.germain2@canada.ca (I.G.); martin.mondor@canada.ca (M.M.); sebastien.villeneuve@canada.ca (S.V.); 2 Sherbrooke Research and Development Centre, Agriculture and Agri-Food Canada, 2000 College Street, Sherbrooke, QC J1M 0C8, Canada; pauline.bilodeau@canada.ca; 3 Saint-Jean-sur-Richelieu Research and Development Centre, Agriculture and Agri-Food Canada, 430 Gouin Boulevard, Saint-Jean-sur-Richelieu, QC J3B 3E6, Canada; marietherese.charles@canada.ca; 4 Biological Collections Division, Agriculture and Agri-Food Canada, 107 Science Place, Saskatoon, SK S7N 0X2, Canada; axel.diederichsen@canada.ca; 5 Guelph Research and Development Centre, Agriculture and Agri-Food Canada, 93 Stone Road West, Guelph, ON N1G 5C9, Canada; qiang.liu@canada.ca; 6 Kentville Research and Development Centre, Agriculture and Agri-Food Canada, 32 Main Street, Kentville, NS B4N 1J5, Canada; shawna.mackinnon2@canada.ca; 7 Agassiz Research and Development Centre, Agriculture and Agri-Food Canada, 6947 Highway 7, Agassiz, BC V0M 1A0, Canada; aime.messiga@canada.ca; 8 Quebec Research and Development Centre, Agriculture and Agri-Food, Canada, 2560 Hochelaga Boulevard, Quebec City, QC G1V 2J3, Canada; noura.ziadi@canada.ca (N.Z.); stephane.gariepy@canada.ca (S.G.)

**Keywords:** corn, beans, squash, pumpkin, maize

## Abstract

For centuries, some Indigenous Peoples of the Americas have planted corn, beans and squash or pumpkins together in mounds, in an intercropping complex known as the Three Sisters. Agriculturally, nutritionally and culturally, these three crops are complementary. This literature review aims to compile historical foods prepared from the products of the Three Sisters planting system used in Indigenous communities in the region encompassing southern Quebec and Ontario in Canada, and northeastern USA. The review does not discuss cultural aspects of the Three Sisters cropping system or describe foods specific to any one Indigenous group, but rather, gives an overview of the historical foods stemming from this intercropping system, many foods of which are common or similar from one group to another. Some of the methods of food preparation used have continued over generations, some of the historical foods prepared are the foundation for foods we eat today, and some of both the methods and foods are finding revival.

## 1. A Brief History

Corn (*Zea mays*) evolved from wild Balsas teosinte (*Zea mays* subsp. *parviglumis*) in modern-day lowland Mexico where it was brought under cultivation and domesticated beginning around 9000 years ago [1,2]. Archaeological remains provide evidence that corn agriculture was brought to the southwestern United States and the Colorado Plateau about 4000 years ago [3] and spread to dominate food production systems throughout much of the Americas by the beginning of European colonization in the 15th century [4,5].

Unlike corn, beans (*Phaseolus vulgaris*) show few changes through 9000 years as domesticated crop plants and those dug from ancient middens in Mexico bear a striking resemblance to modern varieties [6]. About 6000 years ago in the Mexico lowlands, corn and beans were planted together in the same fields [7]. Generally, climbing varieties were intercropped, whereas dwarf varieties or bush beans were monocropped [8]. The common bean was cultivated as a food crop in North America for at least five centuries before European contact [6].

The bottle gourd (*Lagenaria siceraria*) has also been grown in the Americas for at least 9000 years [1]. Like beans and corn, members of the cucurbitaceae family (*Cucurbita* spp.), gourd, squash and pumpkin, also came from Central America and have been grown for at least 5000 years in North America [9]. Squash arrived via the commercial routes of the Great Lakes and in the northeastern USA, the egg gourd and other pepo gourds appeared with corn during the Owasco period, that is, from about 1000 to 1300 AD [6].

Some Indigenous Peoples of the Americas planted corn, beans and squash or pumpkins together in mounds, in an intercropping complex known to some as the Three Sisters. Corn provided support for beans, beans provided nitrogen through nitrogen-fixing rhizobia bacteria that live on the roots, and squash and pumpkins provided ground cover to suppress weeds and inhibit evaporation from the soil [2,10]. While the origins of the Three Sisters complex are unknown, veneration of the Three Sisters appears in the earliest accounts of European explorers and missionaries in North America. As described by Lewandowski [6], from its earliest appearance in written records, the Three Sisters complex was not simply an agricultural strategy or technology, but a cultural complex, complete with stories, ceremonies, technology, customs and etiquette.

Archaeological evidence dates the adoption of the Three Sisters complex in North America to 1070 AD [6]. The complex was adapted to local conditions over the 500 years before contact with Europeans, so much so that it was the dominant food plant association of every nation practising agriculture in the northeast USA as well as in several other parts of North America, including southern regions of Quebec and Ontario, Canada. The Indigenous Peoples practising Three Sisters agriculture in the northeastern USA and southeastern Canada included those from at least fifteen nations [11].

The integration of the cultural complex surrounding the Three Sisters is unknown and may have been imported whole, with the plants, and adapted to other cultures already in place. Regardless of the uncertainty of its integration, earliest European explorers and missionaries found the complex in full bloom, culturally and agriculturally [6]. Indeed, in 1605 Champlain describes seeing corn raised in gardens, with three or four kernels planted in mounds three feet apart and into which three or four beans were also planted so that they grew, interlacing with the corn and keeping the ground free from weeds [12]. He also described seeing many squash and pumpkins. Field size is suggested to have ranged from a few acres to several hundred with early documents supporting some significant garden sizes [6]. In a village on the Hudson river in 1609, Henry Hudson reported seeing enough dried corn to load three ships [13]. Denonville raided Seneca villages in 1687 destroying, by his count, 400,000 minots of corn, equivalent to about 13,600 m^3^ [14]. In New York state in 1779, Major John Burrowes wrote of passing a 100 acre field of produce including corn, beans, squash and witnessing the destruction of 150 acres of corn crops, an estimated 60,000 bushels of corn and 2000–3000 bushels of beans (about 2100 m^3^ and 70–100 m^3^, respectively) [15]. Estimates of crop acreage for the Wendat (Huron), who like other Haudenosaunee (Iroquoian) were farmers who supplemented their diet with hunting and fishing, suggest that a third of an acre per person provided for sustenance [16]. Note that to avoid confusion, in the current review the names of Nations assigned by the authors at the time of the cited reference are given in parentheses following the actual Nation, and this format is used hereafter where discrepancy between cited and accepted names are found. A typical Wendat village was estimated to have from 900 to 1600 people [17] and the total population of the Wendat at the time of European contact has been estimated at 20,000 to 40,000 people [16]. In addition to providing for their own needs, Wendat also cultivated corn on a large scale to exchange for furs and other commodities with neighbouring peoples [8].

Given the sizable crops and the cultural significance of the Three Sisters complex to the agricultural Indigenous communities for centuries in the northeastern USA and in southern Quebec and southern Ontario in Canada, corn, beans and squash have historically featured prominently in the diet in these regions. Indeed, Heidenreich [16] estimated the Wendat diet was 65% corn, 15% beans, squash and pumpkins, 10–15% fish, and 5% meat. Mention of foods stemming from the products of the Three Sisters date back as long as reports of the growing system itself. In 1535, Cartier [18] cruising west along the Saint Lawrence River wrote that he was greeted with gifts of fish and corn bread. In 1615, Champlain [19] reported visiting four villages on the shores of Lake Huron where he feasted on corn, squash and fish, writing specifically of several methods of preparation of corn.

The aim of this review is to consolidate literature on historical foods prepared from the products of the Three Sisters planting system used in Indigenous communities in the region encompassing southern Quebec and southern Ontario in Canada, and the northeastern USA. The review does not aim to discuss cultural aspects of the Three Sisters cropping system or describe foods specific to any one Indigenous group, but rather, aims to give an overview of the historical foods stemming from this system, many of which are common or similar from one group to another.

## 2. Terms

### 2.1. Corn vs. Maize

The terms “maize” and “corn” are often used interchangeably, making for some confusion as to the differences between the two and which term should be used. Corn is primarily used in the USA, Canada, Australia and New Zealand to describe crops of the *Zea mays* species, whereas maize is primarily used in English elsewhere. Maize is the preferred term in formal, scientific, and international usage because it refers to *Z. mays* whereas in many countries, corn is synonymous with the leading cereal grain, such as in England and Scotland where it may refer to wheat and oats, respectively [20,21]. In the culinary context, maize is rarely used for food items prepared using maize grains, so that maize refers to what is yielded from the field, but corn refers to the food on the plate. Given that the focus of this review is on foods and food preparation, the term corn is used throughout the current review.

### 2.2. Squash, Pumpkin and Gourd

The history of some terms used for certain members of the family and genus *Cucurbitacaeae Curcubita* have been reviewed by Sturtevant [22] and Whittaker and Bohn [23]. Sturtevant [22] writes that “pumpkin” designates fruit that are palatable and round, or nearly so, the name deriving from the Latin *pepo* for a round or enlarged fruit, such as a watermelon. Most pumpkins are used when they are fully enlarged and mature. In contrast, “squash” designates fruits that are palatable, but not round. Squash can be used when immature or mature. Finally, “gourd” designates fruit that are generally unpalatable, often small, and sometimes quite bitter. These definitions appear relatively simple, but are not without fault and a half century later, Whittaker and Bohn [23] assigned definitions in agreement with usage, rather than incorporating them into botanical terminology. It was suggested that the term “pumpkin” should be defined as the edible fruit of any species of *Cucurbita* utilized when ripe as forage, as a table vegetable or in pies, but not generally served as a baked vegetable. “Squash” was defined as designating the forms of *C. Pepo* that are used immature, all baking varieties of *C. maxima* and the cushaw forms of *C. moschata* which are used mature. In addition, “summer squash” were defined as the edible fruit of any species of *Cucurbita*, commonly *C. pepo*, utilized when immature as a table vegetable and “winter squash” as the edible fruit of any species of *Cucurbita* utilized when ripe as feed for livestock, as a table vegetable or in pies, and suitable for baking.

Given these somewhat confusing attempts at defining “pumpkin” and “squash”, it is not surprising that these terms appear to be used interchangeably to describe the same produce in many of the references cited in this review. Therefore, unless both terms are mentioned, in the current review “squash” is used throughout to refer to species of *Cucurbita*.

### 2.3. Historical or Traditional

In this review, the term “historical” has been used rather than the term “traditional”. A typical dictionary definition of a tradition is a belief, custom, or way of doing something that has existed for a long time among a particular group of people [24]. While there is not an internationally accepted definition of “traditional knowledge”, the World Intellectual Property Organization [25] has provided a definition of “knowledge, know-how, skills and practices that are developed, sustained and passed on from generation to generation within a community, often forming part of its cultural or spiritual identity”. So, how far back in time constitutes a tradition? Should it be implied from such definitions that the tradition is still relevant or in use today? Accounts, such as that of Parker [26] and Waugh [8] who published a great detail on Iroquois foods and food preparation included historical references, interviews with people who spoke of their memories which may have been more than 50 years earlier, and documents contemporaneous at that time. Given that these books were published more than 100 years ago, are all of the recipes cited “traditional” today, even if some were recent at that time?

A prime example of the complexity of the term “traditional” can be found in bannock in Canada. At its origin, the term bannock was used in the British Isles encompassing all sorts of unleavened flat breads made from a variety of grains [9]. Prior to contact with Europeans, some Indigenous Peoples of Canada had forms of starch-based unleavened bread-like foods, starch being from corn or bracken (*Pteridium* spp.) rhizomes, the underground stems of ferns [27]. With the influence of Scottish and Irish colonists, these bread-like foods came to be known as bannock (or banique in French-speaking Canada). Nowadays in Canada, the term bannock for many is considered a traditional Indigenous food, regardless of the origin of the term or that the bannock of today is most often made with wheat flour and is often leavened. Therefore, when does a custom or method become a tradition? Additionally, does it remain a tradition, even when it has evolved to the point that the original method or custom is no longer recognisable?

The recipes and methods described in the current review are those found in the literature, some of which pre-date colonisation, but many of which have evolved, particularly since European contact and the tools and ingredients this contact brought. Most of the very early accounts are from foreigners on short visits to a given area or community, sometimes even simply notes taken while passing by. While outsiders views could lead to questions of interpretation, corroboration among early accounts and with more recent studies would suggest some accuracy in the reporting. Some of the recipes may still be in use today, but many are no longer, and hence this is a review of literature on historical recipes using produce from the Three Sisters complex. Therefore, for the purpose of this review, the term “historical” is used.

## 3. The Three Sisters

### 3.1. Corn

In 1916, Waugh [8] noted that most of the early writers who dealt with ethnological topics described the varieties of corn, very loosely and inaccurately. In 1588, Hariot [28] stated that there were some white, some red, some yellow, and some blue corn. In 1705, Beverly [29] distinguished four sorts of corn of which two were early ripe and one of these was not much larger than the handle of a case knife with a stalk between three and four feet high. The other two were late ripe and distinguished by the shape of the grain only, with one looking as smooth and as full as early ripe corn, called flint corn, and the other with a larger grain that looked shrivelled with a dent in the back of the grain as if it had never come to perfection, called the she-corn. In 1812, Pinkerton [30] described red, white, yellow, blue, green, black, some speckled and some striped corn. Additionally, in 1851, Morgan [31] mentions white, red and white flint corn.

While Parker in 1910 [26] reported five types of corn distinctions; soft, flint, sweet, pop and pod, Waugh [8] considered that all the corn varieties reported were sub-divisions of the single species *Zea mays*. Lewandowski [6] noted that while a number of varieties of corn were cultivated, it is probable that they were all mutants of eight-rowed Northern Flint corn. Genetic information verifies that certain Northern Flints were selected for high sugar, low oil and high starch contents or heavy seed coat. Although the use of sweet corn was not as important in the diet as hominy or flour corn, in 1884, Sturtevant [32] listed thirty three sweet corn varieties. Pop corn was also a special group of flint corn of which 25 varieties were recognised [8]. And podded corn was suggested a very primitive form of corn as described by Bauhin in 1623 [8].

Although Parker [26] and Waugh [8] used different sources to collate information on corn varieties, there is much overlap in their findings. Pop corn varieties included Red, White, Red Pearl, Rice or Toothed, Smooth or Pearl, Early, Medium and Late. Reported sweet corn varieties were Sweet Puckers, Black Sweet, Sweet and Short-ears. Flint corn varieties included Hominy or Flint, Long-eared, Yellow, Purple and Variegated (Calico). Additionally, soft (flour, starch or bread) corn varieties included Tuscarora or White or Squaw, Short-eared Tuscarora, Purple, Red, Variegated (Calico), Short White, Light Yellow (twelve row) and Black.

More recently, a consistent taxonomic review of the various types of *Zea mays* by Fritsch [33] distinguishes seven convarieties that encompass the different corns described in earlier studies: *Z. mays* convar. *amylaceae* (soft corn); convar *aorista* (intermediate between flint and dent types); convar. *ceratina* (waxy corn); convar. *dentiformis* (dent corn); convar. *mays* (flint corn); convar. *microcarpa* (popcorn) and convar. *sacharata* (sweet corn).

### 3.2. Beans

Cultivated beans were mostly of the genus *Phaseolus*, considered to have been indigenous to South America [8]. The common bean, *P. vulgaris*, was cultivated as a food crop in North America for at least five centuries before European contact [6]. In the Jesuit Relations [34] written in the 17th and 18th centuries, many references to crops of beans are found and the accounts of most early writers make reference to the many varieties of beans observed. For example, in 1545 Cartier [18] noted beans of all colours, in 1705 Beverly [29] mentioned a large variety of beans, all of a kidney shape, and in 1675 Josselyn [35] wrote that many are variegated, some are much bigger than others, some white, black, red, yellow, blue spotted, as well as Bonivis, Calavances and kidney beans. In 1908, Harrington [36] observed nine or more varieties of beans that varied in size from that of a small pea to a large lima bean, and were of varied shape (globular, flat, long and cylindrical or ordinary bean-shape) and colour (solid blue, brown, yellow, blotched, striped or speckled with reddish or bluish tints). Each variety had its own name, and some were considered especially valuable for certain purposes.

In 1916, Waugh [8] discussed collecting over 60 different varieties of beans of which 27 are described in detail with photographs. It was also noted that beans were classified as bread beans and soup beans, as well as cranberry beans (a short, round type), but classifications varied with individual preference. Based on the reports of early bean varieties from a number of sources [8,26,37,38], Lewandowski suggests a list of 23 varieties that could have been used in the Three Sister complex: White Cranberry, Dwarf or Pole Red, Cranberry, Case Knife, Creaseback, Cutshort or Cornhill, Lazy Wife, Yellow Cranberry, China Red Eye, Navy, Sulfur or Eureka, White Kidney, White Marrow, Yellow Eye, Mohawk, Wren’s Egg or London Horticultural, Wampum, Wild Goose, Ground Bird, Feejee, Indian Chief, Red Face, Round Yellow Six Weeks and Long Yellow Six Weeks [6].

### 3.3. Squash

Many varieties of *Cucurbitaceae*, including pumpkin and squash, were cultivated and are mentioned frequently in historical documents [18,28,29,34,35,39]. Lewandowski [6] reviewing various sources of information notes that squash, pumpkins and gourds of *C. pepo* were the most common and cites old *C. pepo* varieties of Scallops, Vegetable Marrow, Crookneck, Small Sugar and Connecticut Field pumpkins, *C. moschata* varieties of Striped Crookneck and Winter or Canada Crookneck and *C. mixta* varieties of Green-Striped and Golden Cushaw.

## 4. Nutrient Complementarity

Estimations of the Wendat diet suggest that 80% was comprised of produce from the Three Sisters complex including 65% corn or about 600 g/person per day [16]. As for the cultural and agricultural aspects of this complex, the nutritional quality of the three components are complementary. Corn is a source of carbohydrates (74%), protein (9.2%) and polyunsaturated oils (2%), but the protein is lacking in the amino acids, lysine and tryptophan [6]. In addition, alkaline processing (nixtamalization, see Section 6.1.4) results in a loss of some oil and protein. However, the alkaline treatment decreases the availability of zein which reduces the relative deficiency of lysine and tryptophan, making for a better balanced protein in the essential amino acids [40]. Beans also provide lysine and tryptophan. Squash is a source of carbohydrates and sugars, and if the seeds are consumed, more protein is added to the diet [6]. All three vegetables are high in fibre and together, provide a range of minerals and vitamins, as well as choline, for the human diet, including vitamins A, B1, B2, B3, B5, B6, B9, C, and E, calcium, phosphorus, magnesium, sodium, potassium, sulphur, iron, manganese, copper, zinc and selenium [41].

It should be noted that modern varieties of sweet corn have a much higher sugar content than traditional varieties and contain less protein, beta-carotene and anthocyanins, the latter being largely responsible for the red, purple and blue colours of kernels. In recent years, interest in reviving ancient coloured sweet corn varieties or developing new pigmented varieties characterized by high contents anthocyanins is increasing due to the potential functional properties of these pigments (as reviewed by Di Gioia, Tzortzakis, Rouphael, Kyriacou, Sampaio, Ferreira, and Petropoulos [42]).

In addition to the complementarity of the nutrients of the Three Sisters produce, the intercropping system yields more energy (12.25 × 106 kcal/ha) and more protein (349 kg/ha) than any of the crop monocultures or mixtures of monocultures planted in the same area [43]. Indeed, Mt Pleasant found that the Three Sisters produced two to four times more energy per given area than monocultures of bean and pumpkin, and slightly more energy than corn, and similar findings were observed for protein [43]. It was also reported that the Three Sisters provided energy for 13.42 people/ha and protein for 15.86 people/ha [43]. While the corn monoculture was similar, with energy for 13.03 people/ha and protein for 14.05/ha, the other monoculture mixtures supported many fewer people, ranging from 7.15 to 11.25 people/ha for energy and 10.64 to 13.05 people/ha for protein.

## 5. Methods of Storage

After harvesting, but prior to preparation for consumption, much of the produce from the Three Sisters complex underwent preparation for storage. These preparation steps and types of storage would have had an impact the primary ingredients.

### 5.1. Corn

In 1632, Sagard [44] described that when the corn plant died off, the ears of corn were harvested, bound together by the upturned leaves and arranged in bundles that were hung throughout the houses. Four husks were usually left on each ear for braiding into a string of five spans in length [8]. A century later Lafitau [45] similarly described that corn was dried on long poles arranged around a fire, the smoke from which blackened the grain a little, and on the porches and exterior vestibules of the cabins. When the grains were very dry, the kernels were removed from the cob, cleaned and placed in large bark barrels or containers (5–6 feet high) which were stored on the porches or in the corners of the houses [19,34,44,45]. In addition, granaries made of bark in the form of towers were built on high ground [45]. The bark was pierced on all sides to allow air to penetrate and prevent the grain from molding. A twelve-foot diameter round structure constructed as an attachment to a house was identified as a granary at an archaeological village site dated 1380 AD in New York State [6]. Only corn reserved for planting was left drying in the smoke. Waugh [8] noted that the size of these containers might be envisaged based on a citation in Jesuit Relations [34] of two bins which held at least 100 to 120 bushels (about 3.5 to 4.2 m^3^).

Sagard [44] and Harrington [36] describe that ears of corns that were not quite ripe were roasted by holding them on a stick supported by two rocks over a fire, and turning them from side to side or putting the ears in a heap of sand that was heated underneath a fire. Once sufficiently roasted, the kernels were detached and spread on bark to dry in the sun. Sagard [44] noted that the dried kernels were mixed with a third or a quarter of beans (flageolet) and stored in barrels, whereas Harrington [36] wrote of barrels of corn only. Waugh [8] wrote that corn that was firm but still milky was boiled or roasted and dried, and the kernels were removed for winter use. Additionally, Onion [46] reported that green corn was boiled then parched briefly over hot coals while still in the husk, the ears were then husked and thoroughly dried in the sun, after which the kernels were stripped from the cob. Much of the original green corn flavour was restored by soaking in water prior to use [47]. Onion [46] explains that this parched green corn was able to be kept for long periods of time in earthen pits without spoiling, whereas the corn harvested in September and simply dried would not keep for as long.

In 1603, Champlain [48] observed the pit method of storage, in which trenches were excavated to a depth of five to six feet, on a dry, sandy slope, and corn grain in bags made of braided grass, was covered with three to four feet of sand. More than two centuries later, Morgan [31] described that surplus dry and charred green corn was stored in caches and that pits of charred corn were still found at ancient settlement sites at this time. A pit was excavated and lined with bark on the bottom and sides, the corn was deposited in the pit and a water tight bark roof was constructed over the pit then covered with earth.

Kalm [49] also described a similar pit method in 1771, but with a couple of notable differences. In addition to the pit being lined with bark, Kalm also mentions that a grass (*Andropogon bicorne*) “supplies the want of bark”. Furthermore, he notes that the “ears of corn are thrown into the hole and covered with a considerable thickness of the same grass” before being covered with earth. This author does not mention the corn being dried or the kernels being removed from cobs, instead the storage pits are described in the same mention as reaping the corn. Kalm [49] does note that the corn stored extremely well in these pits.

Lewandowski [6] notes that the pits were lined with a base of sand and gravel for drainage, ranged from three to six and a half feet deep and from three to four feet in diameter, and could contain seven to ten bushels (about 0.25 to 0.35 m^3^). In 1916, Waugh noted that the storage of corn in pits was no longer practised [8].

Finally, in 1910 Parker [26] mentioned that corncribs had little been improved since European contact. A photograph of a corncrib at the time of publication illustrates a means for storage of whole ears of dried corn, with or without the husk.

### 5.2. Beans

Little is reported on how beans were stored historically. Sagard [44] described mixing the dried kernels of corn that was not quite ripe with a third or a quarter of beans (flageolet) for storage in barrels (Section 5.1). Waugh [8] wrote that green beans in the pod were prepared by boiling, drying in evaporating baskets or on a flat board, and storing in a bag or barrel. Additionally, Lewandowski [6] mentioned that shelves above and below the sleeping bunks were often used to hold elm bark containers of dried foods (parched corn, shelled beans and sun-dried or fire-dried squash).

### 5.3. Squash

Jesuit Relations [34] observed that squash often formed the principal food in certain seasons. It was noted that squash was cut into strips and placed in evaporating trays, or strung on cords suspended near the fireplace until dry, then stored. Furthermore, squash were said to have been placed in storage pits and dug out as occasionally required. Lafitau [45] described that the storage pits described above for corn, were also used for the preservation of garden products, including squash and pumpkin. However, unlike for corn, in 1916 the storage of squash and pumpkin in pits was still practised [8]. Hard squash were raised specifically for storage in pits [6]. Jesuit Relations [34] testified to their hardness saying that it required a hatchet to open them and that they lasted 4–5 months.

## 6. Food Preparation

Historical foods prepared using the produce from the Three Sisters intercropping system as the principal ingredient are summarised in Table 1 and further detailed in the following sections.

Prior to European contact, foods were cooked by boiling in pottery containers that were placed in or over a fire, adding hot stones from a fire to foods in bark or wooden bowls, baking on a flat stone, roasting or cooking in the red hot embers and broiling on spits or sticks stuck into the ground before the fire [8]. Foods that were cooked in the embers were often first covered with a layer of dead coals, then with a layer of hot coals [46]. Pits were also dug into the sides of banks of clay deposits, fire built in the pits, the coals removed and corn, squash, roots and other foods baked by covering with hot ashes [26]. After European contact, brass or copper kettles eventually replaced pottery containers or bark or wooden bowls.

### 6.1. Corn

#### 6.1.1. Corn on the Cob

The corn was picked when the kernels were firm, but still milky. The ears were boiled in the husk for about 30 min [8] or husked and roasted over a fire or in the ashes, then eaten on the cob [34]. Waugh [8] mentions that at the time of publication (1916), butter and salt were often added.

Lafitau [45] noted that when corn is soft and almost milky, it is crushed slightly without separating it from the cob and eaten. Jesuit Relations [34] noted that raw corn was eaten when the rations were very short and consisted solely of freshly picked corn. This statement lead Waugh [8] to suggest that historically, raw corn was eaten during emergencies or when lack of time prohibited further preparation.

#### 6.1.2. Popcorn

Popcorn was simply popped by throwing it in the hot coals in an open fireplace, stirring quickly and pulling it out as it popped [8]. In 1603, Champlain [48] remarked a particular type of corn that he named as “blé-fleuri” due to the fact that when it was heated, it burst open resembling a flower, likely popcorn. He also noted that this surpassed all other corn in terms of taste. Popcorn was eaten as is or used as the base for a number of dishes.

#### 6.1.3. Fine Flour or Parched Corn

In 1632, Sagard [44] described that dry corn was toasted in a mix of embers and sand, pounded and a fine flour was separated from coarse grains by fanning with bark. Corn was sometimes pounded with dried fruit or chopped meat, or if for eating raw, maple sugar [8,36]. However, sugar was not used for hunters or athletes. Fine flour could be eaten dry in small quantities, soaked in warm or cold water, added to water as a beverage, or cooked in stews or soups [8,19,26,29,44]. Bartram in 1751 noted that a quarter of a pound (about 113 g) of this flour (mixed with sugar), in a pint (about 473 mL) of water was a hearty travelling dinner [50]. Harrington noted that, while parched corn was in former days an important article of Iroquois diet, it was at the time of writing (1908) used mainly at certain ceremonial functions [36].

#### 6.1.4. Hominy

Hominy is the term used to describe dried corn kernels that have undergone a process called nixtamalization. Generally, nixtamalization of corn involves soaking and cooking the corn in an alkaline solution, usually lime water but sometimes wood ash lye (a strong alkaline liquor leached from wood ashes and rich in potassium carbonate), washing, and then hulling [54,55]. The strong alkalinity of lime and ash help the dissolution of hemicellulose and thereby the loosening the pericarp (hulls) from the corn kernels. During the nixtamalization process, some of the corn oil is broken down into emulsifying agents (monoglycerides and diglycerides), while bonding of the proteins to each other is also facilitated. The divalent calcium in lime acts as a cross-linking agent for acidic side chains of polysaccharides and proteins. As a result, while cornmeal made from untreated ground corn is unable to form a dough on addition of water, the chemical changes with nixtamalization allow dough formation. Nixtamalized corn has several benefits over unprocessed corn, being that it is more easily ground, has improved nutritional value, flavor and aroma, and reduced levels of mycotoxins.

Ripe corn was usually hulled for cooking, either physically by pounding, or chemically by boiling in lye [8]. Harrington [36] detailed the process explaining that shelled flint corn was placed in boiling water, simmered until well swollen, and then sifted hardwood ashes were added at a proportion of about one quart of ashes to a gallon of water (equivalent to about a 1:5 ratio). The mass was boiled until the black pips of the corn loosened and were seen floating about, and the hull slipped easily when the grain was rolled between the fingers. The mass was poured into the hulling basket and drained, then put into fresh water, or a running stream, basket and all, and vigorously shaken and stirred until the hulls and pips had floated away and the corn was sufficiently washed. The corn was then added to fresh boiling water, rapidly boiled until fairly soft, and washed to remove the last traces of lye. At this stage the hulling process was complete. Waugh wrote that it could take up to 90 min for the hulling process, which included about 45 min for the first boiling, including the lye, and 30 min for the second [8]. However, Briggs [56] in an extensive review on hominy explained that accounts of the time taken are highly variable, ranging from a couple of hours to overnight for soaking in an alkaline solution and anywhere from one to twelve hours boiling time.

A variant of the process was noted in which the ashes were first boiled in the water [8,26,56]. Only when the water had lost its slippery feel and acquired a sharp biting taste, was the corn added to the boiling lye solution. Waugh [8] noted that sifted hardwood ashes were added to a pot of water in the proportion of about one double handful to three quarts of water (about three litres) while Parker [26] described that the lye must be strong enough to bite the tongue when tasted. Harrington’s [36] informant also specified that while the first boiling ought to be slow, the second should be fast. The corn was then ready to use whole, for example, in soups or to pound if required for bread-making. In fact, most foods that could be made from un-hulled corn, could also be made from hominy [46].

#### 6.1.5. Breads and Pastes

In the mid 1600’s, Champlain [53] and Sagard [44] both described baked bread made from dried corn or hominy. If dried corn was used, first it was boiled a little in water, then wiped and dried. In the early autumn, newly ripened corn that had not been dried could also be used [8,36]. The washed dried corn, hominy or fresh corn was ground, beaten to a paste with cold, warm or boiling water, formed into loaves that were sometimes wrapped in corn husks, and cooked in hot embers or on flat stones [34]. Adair [52] also describes a variation in which the paste loaf, first steeped in hot water, was put over the cleared hearth, and an earthen basin placed above it with embers on top. Baked breads required about 45 min for cooking, often turning about half way through. When cooked, if not wrapped, the loaves were washed in cold water to remove ashes or cinders. The bread was eaten fresh or dried, broken into small pieces and stored for winter [36]. When needed, the corn bread pieces were boiled soft and eaten with meat. Beans, small fruits, such as, strawberries, blueberries, raspberries and blackberries, squash that had been boiled to a thin mush, or maple sugar were sometimes added to give taste to an otherwise bland bread. Beans were cooked first in a small pot and then gently mixed into the corn paste so as not to break them up.

A variation of baked bread is described by Sagard in 1632 [44] in which, before the corn plant had died off and the ears had become dry, women, girls and children collected the ears of corn and removed the kernels from the cob with their teeth, spitting them into large bowls. The kernels were ground and the paste wrapped in leaves to cook in ash. It was reported that of the breads, this “masticated” bread was the most esteemed.

Another variation is described in which a kettle was lined with basswood leaves, three leaves deep [26]. Green corn scraped from the cob was beaten to a paste, and placed on top of the leaves filling the kettle up to two thirds full. The paste was then covered by another three layers of leaves, then cold ashes to a fingers depth. A small fire was lit under the suspended kettle and glowing charcoal placed on the ashes on the top. The small fire was maintained and coals on the top replaced three times, then left overnight. In the morning, the ashes and leaves were carefully removed and the baked corn bread dumped out. The bread was eaten fresh with grease or, in more recent times butter, or sliced and dried in the sun over several days for storage for winter. The stored bread was boiled in water for about half an hour before eating.

Boiled bread used the same paste as baked bread [8,36]. However, rather than cooking as one large loaf, lumps of the paste about the size of a double handful were broken off, formed into balls and then slapped back and forth between the palms using a rotary motion to form a round disk about 3.8–4.5 cm thick and 18 cm in diameter. The cakes were slid into a pot of boiling water and supported on edge until all were in the pot. When partially cooked, the cakes were rotated a little. An hour was usually required for cooking, indicated by a tendency for the cakes to float or when steam was given out equally all over when a cake was lifted out. In 1916, Waugh [8] describes that this boiled bread could be sliced and eaten hot or cold, with butter, gravy or maple syrup, or as reported from one of his informants, fried in butter.

Parker [26] described a bread boiled in corn leaves, stating that in order for the paste to have the proper consistency, the corn should be too hard for boiling and eating on the cob. In 1632, Sagard [44] also wrote of a corn leaf-wrapped boiled bread, but this was formed into two balls joined together. In the early 1900’s, Harrington [36], Parker [26] and Waugh [8] all explained that the dumbbell shaped bread was wedding bread. For all of these wrapped breads, ripe corn was shelled, pounded and sifted without the hulling process. Berries and sometimes squash that had been boiled to a mush, were mixed with the meal, which was made into bread and boiled for 45 to 60 min. Wedding bread was made as round loaves or in double packages by tying a number of corn husks at one end, filling the husks with paste, and tying at the other end and in the middle [8]. Wrapped bread was eaten with sunflower or bear oil, and in more modern times, bacon fat or butter [26]. If no beans or berries were added, these breads could be dried for use in winter [26].

Yet another form of boiled bread was dumplings made from cornmeal [8]. The meal was mixed with boiling water to a stiff paste and moulded between the hands to form balls that were dropped into boiling water or were boiled with venison, game birds or other meats, requiring 30 min to cook.

One method of bread making using fried green corn is mentioned by Parker [26]. Green corn was scraped from the cob, pounded to a paste and either patted into cake or tossed in a basket to make a loose light mass. The paste or mass was fried, and it was noted that bear oil was especially good for frying.

#### 6.1.6. Soups, Stews, Mush and Pudding

A large proportion of foods were evidently of liquid nature, substantiated by numerous references to soups and broths, which were easily prepared and nourishing while answering the purposes of a beverage [8]. As noted, many of these dishes could be prepared as either savoury or sweet dishes.

A soup was made from the liquor left after boiling corn bread [8]. The coarser particles left after grinding and sifting were sometimes added, as was maple sugar as a sweetener. In 1916, Waugh’s informants reported using sweet milk, buttermilk or granulated sugar as sweeteners and salt and pepper as seasoning [8].

Green corn soup or succotash [8,51] made from corn that was not quite ripe, the kernels of which had been roasted, dried, and stored with beans, was described by Sagard in 1632 [44]. The corn and bean mix was boiled with a little meat or fish, fresh or dry, if any was available. Jesuit Relations [34] also write of a corn and bean soup cooked in clear water without seasoning. Waugh [8] details soup made from corn when it is firm, but not yet ripe, that had been boiled, or boiled and roasted (whole ears over the fire or kernels over embers) then dried. This corn was also cooked with meat. In addition, the same soup could be made from fresh unripe corn and beans, to which other ripe vegetables were added, such as peas and squash. This soup was sweetened with maple sugar or seasoned with salt and butter.

Firm, but not yet ripe corn that had been nixtamalized using wood ash (Section 6.1.2) was also made into soup [8]. The nixtamalized corn, whole or lightly crushed with a little water, was boiled with meat or green beans in the pod or with berries and sugar.

While today hominy is the term for nixtamalised corn, Harrington [36] and Waugh [8] detail the preparation of hominy as a simple soup or stew, very often being of little more than cornmeal and water, also called samp or mush. To prepare this version of “hominy”, corn kernels were hulled by pounding with a little water and sometimes a small amount of soda, slowly at first to loosen the hulls and then more vigorously until it was broken into coarse particles. The corn was then sifted and the coarser particles returned to the mortar. While Waugh [8] stated that the coarse portion left after the second sifting was thrown away, Jesuit Relations [34] notes that the coarse grains of flour were cooked in water or with fish, if available. The finer meal was winnowed (air was blown through the meal to remove the chaff) by tossing in a bowl or basket so as to expose the contents as much as possible to the wind. Coarser hulls were often brushed away with the wing of a fowl or bark. From this, the corn was added to boiling water and cooked slowly for about four hours, with frequent stirring and occasional skimming to remove the hulls which still came floating up from time to time [36]. At the end of two hours beans could be added. Waugh [8] noted that the hominy sifter used when pounding was coarser than that used to prepare meal for corn bread. However, Sagard [44] also describes a version of soup using only the fine flour (as described Section 6.2.1 above), added in sufficient quantity to thicken the stew, sometimes with pieces of squash and sunflower seeds added. In 1640, Jesuit Relations [34] mentions seasoning this type of soup with smoked fish.

In another version of this soup, called early hominy, the corn that had ripened, but not dried was used [8,36]. Kernels were shelled and pounded lightly in a mortar so as to crush a little, then added to boiling water. Whole, not quite ripe beans were added and the boiling continued until cooked. To make a pudding, the corn was pounded to a moist meal, then added to already boiled meat, stirring until the corn swelled.

While Waugh [8] wrote that hominy was “sagamité” to the early French, in 1632, Sagard [44] wrote that “ordinary” sagamité was made using flour from raw corn, without separating the fine flour from the coarse grains. The flour was boiled with a little fresh, dried or smoked meat or fish, if available, and sometimes with chopped squash if in season, but most often with nothing at all. Sometimes beans were added after cooking separately so as to keep them whole [8]. To avoid sticking to the pot, the sagamité had to be stirred constantly and after coming to a boil, was immediately eaten. Sagamité was also made with corn that had not been pounded, but this was very tough to cook [44]. In 1639, Jesuit Relations [34] noted that pieces of cinders or a handful of little water flies were sometimes added to season sagamité and it was served with a little seal oil or melted bear or moose fat on top, if available. Furthermore, it was noted that sometimes fish was kept after the fishing season to break into the sagamité during the year, and the more “tainted” the fish, the better. Waugh’s informants described that salmon was hung in the sun until rotten, after which a pointed stick was stuck into the abdomen letting the rotted flesh and other contents run into a pot of the cornmeal mush to cook together [8]. Others described that the hulled corn mush could be eaten with milk and sugar, in the same way as rice or porridge [8].

A variant of these soups was made with squash [8]. Coarse corn meal was boiled to a thin mush. Dried squash was put into water, pounded slightly, and sifted in the coarse hominy basket. The squash was then added to the boiling corn mush and the mix boiled for about 2 h. Corn soup could also contain nut meats of various kinds or sunflower seeds, pounded in a mortar, sifted and added to the soup [8].

Finally, popcorn was also used as a base for mush or pudding and soup or hominy [8]. The corn was first popped (Section 6.1.3), then pounded, sifted, and added to boiling water until it thickened to the desired consistency. As described by Waugh in 1916, pudding was eaten with maple sugar, syrup, sugar, milk, cream or sour milk [8]. Soup or hominy were boiled with meat and seasoned with salt or boiled with maple sugar then cooled and eaten with milk.

#### 6.1.7. Smelly/Stinky Corn

Champlain in 1603 and Sagard in 1632 describe smelly or stinky corn, which was made from ears of corn that had not yet completely dried which were placed in water for two to three months [44,53]. While Sagard described the water as “smelly”, Champlain wrote of placing the corn under the “bourbe”, the deposit that accumulates at the bottom of stagnant water or thick mud of ponds and marshes. Once fermented, the corn was removed from the water and cooked by roasting under hot ashes. Sagard described how the women licked their fingers when handling these stinky ears of corn, as if it were sugar cane, regardless of the incredibly strong, “disagreeable” taste and odour. In the early 1900’s, Parker [26] and Waugh [8] found no recollection of this dish.

#### 6.1.8. Beverages and Sauce

Adair [52] writes that water was rarely drunk. Instead a beverage was made from corn that was pounded until all the “husks” (should this have read “hulls”) came off, then was sifted and fanned, and boiled in large earthen pots. The thinnest part was strained off and mixed with cold water until sufficiently liquid for drinking. Parker [26] noted that in the course of boiling bread, some of the meal on the outside of the cake comes off together with a quantity of starch, and mixes with the water. When the bread is sufficiently cooked, this liquid is poured out in bowls and drunk as tea. Parker also described fine flour from dried corn added to water as a beverage (see Section 6.1.3) and aparched corn “coffee” in which corn was well burnt and parched on the coals, scraped from the cob and boiled about five minutes [26].

The only mention of sauce found is in 1639, when Jesuit Relations [34] wrote that the usual sauce with the food is water, or juice of corn or squash.

#### 6.1.9. Cornstalks

In 1751, Bartram [50] observed that corn stalks were chewed, with the substance spit out after the juice had been sucked out. Almost two centuries later, Waugh mentions that older people who were interviewed remembered seeing this as a means to quench thirst, but classed this as an obsolete food [8]. A syrup is also described as being extracted by boiling or evaporating the juice of young and green cornstalks [26].

### 6.2. Beans

#### 6.2.1. Green Beans in the Pod

Fresh green beans in the pod were cooked in several ways [8,26]. Green beans in pods were cut into pieces and boiled until tender for soup (Section 6.1.6), sometimes with meat, boiled whole without slicing and eaten by drawing the pod through the teeth leaving the strings or fibres behind, or boiled until tender then fried in bear or sunflower oil. Butter and seasonings are noted as being used for all of these preparations. Waugh [8] also wrote of a preparation that was considered very old in which green beans were cooked in their pod with squash that had been cut into small pieces. Additionally, Parker [26] refers to cooking green beans in the pod and when nearly dry, serving them in the shell of a boiled squash.

Green beans in the pod that had been boiled, dried and stored were soaked then boiled to make soup [8].

#### 6.2.2. Shelled Beans

Harrington [36] describing shelled beans, noted that beans were rarely cooked alone. Ripe shelled beans, as a principal ingredient, were boiled with sweet corn, boiled and mashed, or even boiled until soft and then sugar added to make a sweet soup [8,26]. The beans in the first two dishes were sometimes boiled with meat, when available. If the beans had been dried, they were pounded coarsely, soaked in cold water and boiled down to a pudding with meat [26]. Fresh or dried, shelled beans were also frequently mixed into cornbread (Section 6.1.5) or used in soups and stews (Section 6.1.6). Often the beans were cooked before adding to retain some firmness and mixed in at a late stage of the stew or soup preparation so that they remained whole [8].

Fully formed, but not yet ripe beans were also shelled and boiled or used in early hominy soup (Section 6.1.6) [8].

### 6.3. Squash

#### 6.3.1. Boiled Squash

Fresh squash halves were wrapped in basswood leaves and boiled for 2 h after which the leaves were removed and the squash eaten [8]. Fresh squash was also cut into pieces and boiled with green shelled beans or mashed [8]. Likewise, dried squash was boiled, sometimes with meat, or mashed, but was first washed in warm water and soaked to soften or pounded and sifted to a fine meal. Upon eating, deer suet and maple sugar were sometimes added to these preparations. Waugh [8] describes a pudding made by adding sugar and cornmeal to boiled squash.

#### 6.3.2. Baked Squash

In 1636, Jesuit Relations [34] stated that squash was so good that, on being cooked whole under the ashes, it was eaten “as apples are in France”. Waugh [8] writes of cake in which dried squash was pounded, sifted and then soaked in cold water for 60–90 min. It was then sweetened and fat was added. The squash mix was placed in a pan, marked with a knife and baked.

#### 6.3.3. Squash Added to Corn Bread and Soups and Stews

Fresh or dried squash was cut into small pieces, boiled and mashed, then mixed into the paste when making cornbread (Section 6.1.5). Squash pieces were added to hominy soup or sagamité and mashed squash was added to corn-based mush (Section 6.1.6).

#### 6.3.4. Squash Flowers

In 1751, Bartram [50] reported being served a meal from a kettle full of young squash and their flowers, boiled in water with a little meal mixed in. However, in 1916, Waugh wrote that little recollection of the use of squash flowers existed [8].

## 7. Today

As for all socio-cultural practices, foods and their methods of production and preparation have evolved over the years, some beyond recognition, and there are many versions of these historical foods that today use a range of ingredients, adapting to tastes, availability and convenience. A prime example is found in an article on cornbread [57], clearly based on historic boiled recipes, but using canned beans and *masa harina* (flour from nixtamalized corn) for convenience, adding salt for taste, boiling in a pot on an electric stove top, eating with steak and gravy on Sundays as a modern tradition, and frying day-old bread to eat like pancakes with butter and syrup.

The historical cooking methods, while perhaps very time consuming, are basic in today’s world. However, this simplicity translated to relatively healthy foods. While these historical foods are the foundation for many of the foods we eat today, as the tastes of today have evolved so too have the foods, responding to or driving these tastes, not always for the better. Indeed, levels of sugar and/or fat in both the primary foodstuff, such as the corn, and the prepared foods are often significantly higher today than in the historical products. Return to the original foods and methods of preparation are unlikely in most cases, especially given the changes in taste preferences, the desire for convenience and the choice of foodstuffs in today’s market.

However, some methods have continued over the generations and evidence is found in the abundance of websites on all aspects of the Three Sisters planting system and related food preparation, such as a short article on lying corn to make soup and the three or four lyers in the Tuscarora and Six Nations community who have the skill to do this task [58]. In addition, there is a revitalisation of some traditional food and agriculture in North America, such as the Iroquois White Corn Project [59] and the Onondaga Nation farm where Indigenous corn keepers have preserved thousands of historical seeds as a means to help communities recover and reunite with their traditional foods [60]. Indeed, the agricultural sustainability of the Three Sisters, like other historical intercropping systems, such as, the planting of turnips with cereals, including rye and barley in northern Europe as described by Tomson, Bunce and Sepp [61], potentially has a place in the permaculture of today. Additionally, included in contemporary initiatives to restore and revive traditional foods and food culture are promotion of healthier versions of some of the modern preparations of these foods using readily available ingredients, such as, the reports published by the USDA Food Distribution Program on Indian Reservations [62,63], Keep the Campfires Burning program (described in [64]), and the Métis Cookbook and Guide to Healthy Living [65]. And, there are those of us who find adventure in incorporating these traditional methods and ingredients in contemporary cuisine, from Indigenous chefs promoting their cuisine to inner-city restaurants to foodies and gardeners in our own homes.

## Figures and Tables

**Table 1:** Historical Indigenous food preparation with produce of the Three Sisters intercropping system as the principal ingredient.

Principal Ingredient	Cooking Method	State of Principal Ingredient	Food	Preparation	Other Additives	Eaten with:	References
Corn	Raw	Fresh corn cobs (soft, milky kernels)	Corn on the cob	Kernels crushed slightly without separating from cob.			[34,45]
Corn	Raw	Juice from corn	Sauce				[34]
Corn	Raw	Cornstalks	Thirst quencher	Stalks chewed to obtain juice.			[50]
Corn	Boiled	Fresh corn cobs (firm, milky kernels)	Corn on the cob	Boiled in the husk.			[8]
Corn	Boiled	Fresh, dry or nixtamalised kernels	Bread	Dry kernels boiled first. Ground, beaten to a paste with water, formed into discs, boiled.	Sometimes beans, berries, squash or maple sugar.	Butter, gravy, or maple syrup, or fried in butter.	[8,36]
Corn	Boiled	Fresh kernels (too hard for boiling and eating on the cob)	Bread	Ground, and sifted, beaten to a paste with water, formed into balls, wrapped in corn husks, boiled.	Sometimes beans, berries or squash.	Sunflower or bear oil. Later, bacon fat or butter. Could be dried for winter.	[8,26,36,44]
Corn	Boiled	Liquid from preparation of corn bread	Soup		Sometimes corn meal or maple sugar. Later, sweet milk, buttermilk, or sugar.		[8]
Corn	Boiled	Liquid from preparation of corn bread	Beverage				[26]
Corn	Boiled	Cornmeal	Dumplings	Mixed with water to make a paste, formed into balls, boiled with meat.	Meat.		[8]
Corn	Boiled	Dried kernels (unripe) that had been boiled and/or roasted	Soup (succotash or green corn soup)	Boiled with beans.	Beans. Sometimes meat, fish, peas, squash, or maple sugar. Later, salt or butter.		[8,34,44,51]
Corn	Boiled	Nixtamalized kernels (firm, unripe)	Soup	Boiled whole or lightly crushed with water.	Meat, green beans in the pod or berries and sugar.		[8]
Corn	Boiled	Nixtamalized kernels	Soup (early hominy)	Lightly crushed, boiled.	Beans (unripe).		[8,36]
Corn	Boiled	Nixtamalized kernels	Pudding or mush	Pounded to a moist meal, added to boiled meat, stirred until swelled.	Meat.		[8,36]
Corn	Boiled	Cornmeal from physically hulled kernels	Soup (hominy)	Boiled.	Sometimes beans, squash, sunflower seeds or fish.		[8,34,36,44]
Corn	Boiled	Flour from fresh corn kernels	Stew (sagamité)	Boiled.	Sometimes meat or fish (fresh, smoked or dried), rotting fish, squash, beans, nut meats, sunflower seeds, cinders or waterflies.	Seal oil, melted bear or moose fat. Later, milk and sugar.	[8,34,44]
Corn	Boiled	Popcorn flour	Pudding or mush	Boiled.		Maple sugar or syrup. Later, sugar, milk, cream or sour milk.	[8]
Corn	Boiled	Popcorn flour	Soup (hominy)	Boiled.	Meat or maple sugar.		[8]
Corn	Boiled	Corn flour from physically hulled corn kernels	Beverage	Boiled, thinnest part strained off and diluted in water.			[52]
Corn	Boiled	Kernels from parched cobs	Beverage	Boiled.			[26]
Corn	Boiled	Cornstalks (young, green)	Syrup	Boiled and concentrated.			[26]
Corn	Baked	Fresh cobs (firm, milky kernels)	Corn on the cob	Husked, baked in ashes.			[34]
Corn	Baked	Dry kernels	Popcorn	Thrown in hot coals.			[8,48]
Corn	Baked	Dry kernels	Flour	Baked in a mix of embers and sand, then pounded to a fine flour. Eaten dry or soaked in water, or used as an additive to soups and stews.			[8,19,26,29,36,44,50]
Corn	Baked	Fresh, dry or nixtamalised kernels	Bread	Boiled if dry. Ground, beaten to a paste with water, formed into loaves, sometimes wrapped in husks, baked.	Sometimes beans, berries, squash or maple sugar.	Eaten fresh or dried for winter. If dried, boiled until soft and eaten with meat.	[8,34,36,44,52,53]
Corn	Baked	Fresh cobs	Bread	Kernels removed with teeth, ground, beaten to a paste with water, formed into loaves, baked in ashes.			[44]
Corn	Baked	Fresh cobs	Fermented corn on the cob	Placed in still water for 2–3 months, baked in hot ashes.			[44,53]
Corn	Roasted	Fresh cobs (firm, milky kernels)	Corn on the cob	Husked, roasted over a fire.			[34]
Corn	Roasted	Fresh kernels	Bread	Beaten to a paste, placed on three layers of basswood leaves in a kettle, covered with three layers leaves then cold ashes, suspended overnight above a fire and charcoal placed on the cold ashes.		Grease. Later, butter. Could be dried for winter. If dried, boiled until soft.	[26]
Corn	Fried	Fresh kernels (green)	Patties	Pounded to a paste, formed into patties, fried.		Bear oil.	[26]
Beans	Boiled	Fresh green beans in the pod	Vegetable dish	Boiled whole.			[8,26]
Beans	Boiled	Fresh green beans in the pod	Vegetable dish	Boiled with squash.	Pieces of squash.		[8,26]
Beans	Boiled	Boiled then dried fresh green beans in the pod	Soup	Soaked, then boiled.			[8,26]
Beans	Boiled	Fresh shelled beans	Vegetable dish	Boiled with sweet corn.	Sometimes meat.		[8,26]
Beans	Boiled	Fresh shelled beans	Vegetable dish	Boiled and mashed.	Sometimes meat.		[8,26]
Beans	Boiled	Fresh shelled beans	Soup	Boiled.	Sugar.		[8,26]
Beans	Boiled	Dried beans	Pudding or mush	Pounded coarsely, soaked in cold water, boiled with meat.	Meat.		[26]
Beans	Fried	Fresh green beans in the pod	Vegetable dish	Boiled whole then fried in bear or sunflower oil.	Bear or sunflower oil.		[8,26]
Squash	Boiled	Fresh squash	Vegetable dish	Halved, wrapped in basswood leaves, boiled.			[8]
Squash	Boiled	Fresh squash	Vegetable dish	Cut into pieces, boiled with beans.	Fresh green beans in the pod.		[8]
Squash	Boiled	Fresh squash	Vegetable dish	Cut into pieces, boiled and mashed.		Sometimes deer suet and maple syrup.	[8]
Squash	Boiled	Dried squash pieces	Soup or mush	Washed, soaked in water to soften or pounded to a meal, boiled.	Sometimes with meat.	Sometimes deer suet and maple syrup.	[8]
Squash	Boiled	Dried squash pieces	Vegetable dish	Washed, soaked in water to soften, boiled and mashed.			[8]
Squash	Boiled	Fresh young squash and squash flowers	Vegetable dish	Boiled with cornmeal.	Cornmeal.		[50]
Squash	Baked	Fresh squash	Vegetable dish	Baked whole in ashes.			[34]
Squash	Baked	Dried squash pieces	Cake	Pounded, sifted, soaked in water, sweetened, fat added, baked.			[8]

## References

[1] Piperno D.R., Ranere A.J., Holst I., Iriarte J., Dickau R. (2009). Starch grain and phytolith evidence for early ninth millennium B.P. maize from the Central Balsas River Valley, Mexico. Proc. Natl. Acad. Sci. USA.

[2] Mann C.C. (2011). 1491: New Revelations of the Americas Before Columbus.

[3] Merrill W.L., Hard R.J., Mabry J.B., Fritz G.J., Adams K.R., Roney J.R., MacWilliams A.C. (2009). The diffusion of maize to the southwestern United States and its impact. Proc. Natl. Acad. Sci. USA.

[4] Kistler L., Maezumi S.Y., de Souza J.G., Przelomska N.A.S., Costa F.M., Smith O., Loiselle H., Ramos-Madrigal J., Wales N., Rivail Ribeiro E. (2018). Multiproxy evidence highlights a complex evolutionary legacy of maize in South America. Science.

[5] Hart J.P., Lovis W.A. (2013). Reevaluating what we know about the histories of maize in Northeastern North America: A review of current evidence. J. Archaeol. Res..

[6] Lewandowski S. (1987). Diohe’ko, The three sisters in Seneca life: Implications for a native agriculture in the Finger Lakes region of New York State. Agric. Human Values.

[7] Kaplan L. (1965). Archaeology and domestication in American Phaseolus (Beans). Econ. Bot..

[8] Waugh F.W. (1916). Iroquois Foods and Preparation.

[9] Kayler F., Michel A. (1996). Cuisine Amerindienne. Un Nouveau Regard.

[10] Pleasant J.M., Staller J.E., Tykot R.H., Benz B.F. (2006). The science behind the Three Sisters mound system. An agronomic assessment of an Indigenous Agricultural System in the Northeast. Histories of Maize: Multidisciplinary Approaches to the Prehistory, Linguistics, Biogeography, Domestication, and Evolution of Maize.

[11] Sturtevant W.C., Trigger B.G. (1978). Handbook of North American Indians.

[12] De Champlain S. (1613). Champlain’s Voyages.

[13] Asher G.M. (1809). Henry Hudson the Navigator: The Original Documents in Which is Career is Recorded.

[14] Serrigny E. (1883). Journal d’une Expédition Contre les Iroquois au Canada en 1687 Rédigé par Le Chevalier de Baugy.

[15] Cook F. (1887). Journals of the Military Expedition of Major General John Sullivan against the Six Nations of Indians in 1779 with Records of Centennial Celebrations.

[16] Heidenreich C.E., Sturtevant W.C., Trigger B. (1978). Huron. Handbook of North American Indians.

[17] Warrick G. (2003). European infectious disease and depopulation of the Wendat-Tionontate (Huron-Petun). World Archaeol..

[18] Cartier J., d’Avezac M. (1863). Bref Recit et Succincte Narration de la Navigation Faites en MDXXXV et MDXXXVI par le Capitaine Jacques Cartier aux Iles de Canada, Hochelage, Saguenay et Autres.

[19] De Champlain S. (1619). Voyages et Decouvertures faites en la Nouvelle France, Depuis L’année 1615 Jusques à la Fin de L’année 1618.

[20] CAB International (2019). Invasive Species Compendium. https://www.cabi.org/isc/datasheet/57417.

[21] Ensminger M.E., Ensminger A.H., Konlande J.E., Robson J.R.K. (1994). Foods and Nutrition Encyclopedia.

[22] Sturtevant E.L. (1890). The history of garden vegetables (continued). Am. Nat..

[23] Whittaker T.W., Bohn G.W. (1950). The taxonomy, genetics, production and uses of the cultivated species of *Cucurbita*. Econ. Bot..

[24] Oxford University Press (2020). Oxford Learners Dictionary. https://www.oxfordlearnersdictionaries.com/.

[25] World Intellectual Property Organization Note on the meanings of the term “public domain” in the intellectual property system with special reference to the protection of traditional knowledge and traditional cultural expressions/expressions of folklore. Proceedings of the Intergovernmental Committee on Intellectual Property and Genetic Resources, Traditional Knowledge and Folklore, Seventeenth Session.

[26] Parker A.C. (1910). Iroquois Uses of Maize and Other Food Plants.

[27] Colombo J.R., Dunne B. (2017). Bannock. The Canadian Encyclopaedia.

[28] Hariot T. (1588). A Briefe and True Report of the New Found Land of VIRGINIA.

[29] Beverly R. (1705). The History and Present State of Virginia, in Four Parts.

[30] Pinkerton J. (1812). A General Collection of the Best and Most Interesting Voyages and Travels in All Parts of the World.

[31] Morgan L.H. (1851). League of the Ho-dé-no-sau-nee or Iroquois.

[32] Sturtevant E.L. (1887). History of garden vegetables. Am. Nat..

[33] Fritsch R. (2001). In Mansfeld’s World Database of Agricultural and Horticultural Crops. https://mansfeld.ipk-gatersleben.de/apex/f?p=185:3.

[34] Thwaites R.G., Jesuit Relations (1897). The Jesuit Relations and Allied Documents: Travels and Explorations of the Jesuit Missionaries in New France, 1610–1791.

[35] Josselyn J. (1675). A Relation of Two Voyages to New-England.

[36] Harrington M.R. (1908). Some seneca corn-foods and their preparation. Am. Anthropol. New Ser..

[37] Jarvis C.D. (1908). American varieties of beans. Bull. Cornell Univ. Agric. Exp. Stn..

[38] Hedrick U.P., Hall F.H., Hawthorn L.R., Berger A. (1931). The Vegetables of New York. I. Legumes, Cucurbits, Corn, Alliums, Asparagus. Part II: Beans.

[39] Brickell J. (1737). The Natural History of North-Carolina.

[40] Bressani R., Scrimshaw N.S. (1958). Lime-heat effects on corn nutrients, effect of lime treatment on in vitro availability of essential amino acids and solubility of protein fractions in corn. J. Agric. Food Chem..

[41] USDA (2020). Food Data Central. https://fdc.nal.usda.gov/index.html.

[42] Di Gioia F., Tzortzakis N., Rouphael Y., Kyriacou M.C., Sampaio S.L., Ferreira I.C.F.R., Petropoulos S.A. (2020). Grown to be blue—antioxidant properties and health effects of colored vegetables. Part II: Leafy, fruit, and other vegetables. Antioxid. Basel.

[43] Mt Pleasant J. (2016). Food yields and nutrient analyses of the Three Sisters: A Haudenosaunee cropping system. Ethnobiol. Lett..

[44] Sagard G. (1632). Le Grand Voyage du Pays des Hurons.

[45] Lafitau J.-F. (1724). Moeurs des Sauvages Ameriquains Comparees aux Moeurs de Premiers Temps.

[46] Onion D.K. (1964). Corn in the culture of the Mohawk Iroquois. Econ. Bot..

[47] Will G.F., Hyde G.E. (1917). Little Histories of North American Indians. Number 5. Corn among the Indians of the Upper Missouri.

[48] De Champlain S. (1603). Des Sauvages ou Voyage Samuel Champlain de Brouage fait en la France Nouvelle l’an Mil Six Cens Trois.

[49] Kalm P., Forster J.R., Translator T. (1771). Travels into North America: Containing Its Natural History, and a Circumstantial Account of Its Plantations and Agricul-ture in General: With the Civil, Ecclesiastical and Commercial State of the Country, the Manners of the Inhabitants, and Several Curious and Important Remarks on Various Subjects.

[50] Bartram J. (1751). Observations on the Inhabitants, Climate, Soil, Rivers, Productions, Animals and Other Matters Worthy of Notice.

[51] Vitart A., Kayler F., Michel A. (1996). “Les manieres de table” Indiennes. Ce qu’en pensaient les Européens. Cuisine Amerindienne. Un Nouveau Regard.

[52] Adair J. (1775). The History of the American Indians.

[53] De Champlain S. (1640). Les Voyages de la Nouvelle France Occidentale, dite Canada Faits par le Sr. de Champlain Xainctongeois, Capitaine pour le Roy en la marine du Ponant, & Toutes Les Descouvertes qu’il a Faites en ce Païs Depuis l’an 1603, Jusques en l’an 1629.

[54] Ramírez-Araujo H., Gaytán-Martínez M., Reyes-Vega M.L. (2019). Alternative technologies to the traditional nixtamalization process: Review. Trends Food Sci. Tech..

[55] Escalante-Aburto A., Mariscal-Moreno A.M., Santiago-Ramos D., Ponce-García N. (2020). An update of different nixtamalization technologies, and its effects on chemical composition and nutritional value of corn tortillas. Food Rev. Int..

[56] Briggs R.V. (2015). The hominy foodway of the historic native Eastern Woodlands. Nativ. South..

[57] Alfred G.T. (2014). Kana’tarokhónwe: Cornbread. Cuizine.

[58] Tuscarora and Six Nations Websites, 1996–2019. By Loren (Lolly) Green 1928–1999. http://www.tuscaroras.com/index.php/traditional-indian-corn-soup-recipe.

[59] (2017). Friends of Gonandagan. https://ganondagan.org/whitecorn/about.

[60] Bleir G. (2019). Indigenous Corn Keepers are Helping Communities Recover and Reunite with their Traditional Foods. https://intercontinentalcry.org/indigenous-corn-keepers-are-helping-communities-recover-and-reunite-with-their-traditional-foods/.

[61] Tomson P., Bunce R.G.H., Sepp K., Agnoletti M., Emanueli F. (2016). Historical development of forest patterns in former slash and burn sites in Southern Estonia. Biocultural Diversity in Europe.

[62] USDA Food Distribution Program on Indian Reservations (2008). A River of Recipes: Native American Recipes Using Commodity Foods. https://pensacolawellness.com/wp-content/uploads/2014/06/River_of_Receipes_Book_Native_American_Observances_Turner_Oct_101.pdf.

[63] USDA Food Distribution Program on Indian Reservations (2013). A Harvest of Recipes: Native American Recipes Using Commodity Foods. https://fns-prod.azureedge.net/sites/default/files/resource-files/HarvestofRecipes.pdf.

[64] Bodirsky M., Johnson J. (2008). Decolonizing diet: Healing by reclaiming traditional indigenous foodways. Cuizine.

[65] Métis Centre, National Aboriginal Health Organization (2008). Métis Cookbook and Guide to Healthy Living.

